# Swedish Sonographers’ perceptions of ergonomic problems at work and their suggestions for improvement

**DOI:** 10.1186/s12891-016-1245-y

**Published:** 2016-09-15

**Authors:** Jenny Gemark Simonsen, Gunvor Gard

**Affiliations:** 1 Division of Occupational and Environmental Medicine, Lund University, SE-221 85 Lund, Sweden; 2 Department of Health Sciences, Faculty of Lund University, SE-221 00 Lund, Sweden; 3 and Department of Health Sciences, Luleå University of Technology, S-971 87 Luleå, Sweden

**Keywords:** Content analysis, Ergonomics, Female, Sonography, Work environment

## Abstract

**Background:**

Sonographers’ perceptions of ergonomic and work-related pain problems at work have so far mostly been researched in quantitative studies by questionnaires. There is a need of experience-based research to deepen the knowledge about how sonographers perceive ergonomic problems at work. Therefore, the aim of this qualitative study was to describe sonographers’ perceptions of ergonomic problems at work, and their suggestions for improvement strategies.

**Methods:**

Twenty-two female sonographers were individually interviewed regarding different aspects of their physical working environment. Content analysis was applied.

**Results:**

The sonographers perceived different ergonomic problems in their working environment, but to offer patient comfort and to obtain the best possible images were often prioritized over working posture. Echocardiography was considered demanding as the examination is performed with little variation in posture. Ergonomic improvements included reducing the manual handling of the transducer, optimizing the adjustability of equipment, and taking the patient’s physique and health into account. As some examinations were perceived to be more ergonomically demanding, variation between examinations was suggested, however, this requires broader skills.

**Conclusion:**

Sonography, especially echocardiography is ergonomically demanding but the improvement strategies suggested were perceived useful and applicable.

## Background

Sonographers constitute a professional group with a high reported prevalence of work-related musculoskeletal pain and discomfort [1], especially in the neck, upper limbs and back [2–6]. Twisted postures, sustained shoulder abduction, a tight hand grip, more than 10 years of working experience and long scanning times each day have been shown to be associated with symptoms and a higher occurrence of work-related musculoskeletal disorders among sonographers (WMSD) [3, 7–11].

Village and Trask reported that sonographers had their scanning arm abducted 30° 66 % of their scanning time [12]. Results by Arvidsson et al. showed that sonographers perceive a high prevalence of hand pain [5]. They also perceive high sensory demands concerning eyesight, precision, attention, focus and control of body movements [5].

A relation has also been found between ultrasonography and WMSD in radiologic technologists [13], and an association has also been reported between twisted postures and physical symptoms in sonographers in obstetrics and gynaecology [14].

Sonography is an important diagnostic tool in daily medical practice [10] with little risk of adverse effects on the patient [4]. Scanning usually takes place in a darkened room, with the patient lying on a table. The sonographer usually sits during the examination, holding a transducer attached to the equipment with a cable, in one hand, while managing the control panel with the other, and observing the images on a screen. Handling the transducer involves static positions of the shoulder, and repetitive, force demanding and precise movements of the wrist and hand [9, 15, 16].

The level of artificial light has to be low to facilitate interpretation of the images on the screen, which may cause visual strain [15]. Examinations are sometimes carried out on the ward with the patient in bed (bedside examination). This is additionally strenuous, as the sonographer must adjust his or her working posture to the bedside situation [17].

Working posture varies depending on the type of sonographic examination being performed. In vascular examinations, for example vein mapping, the handling of the transducer involves diverse movements and postures. In echocardiography the postures and movements are less varied, and the transducer is held in a relatively fixed position. Echocardiography also requires higher grip strength than vein mapping [8]. Greater pressure must be applied to the transducer in corpulent and obese patients undergoing echocardiography [18]. A robot-assisted system in which an arm holds the transducer has been tested, but has not yet been introduced in routine in sonography [19, 20].

The ergonomic challenges of sonography have been known for many years. The Society of Diagnostic Medical Sonography developed industry standards, in 2003, to prevent sonographers from WMSDs [21]. The standards include guidelines for the ultrasound equipment, workload and best ergonomic practices. Horkey and King noted that the sonographers were aware of most ergonomic recommendations but the implementation of these was not satisfactory enough, which depended on among other things lack in organisation and planning, but also on unavailability of adjustable ultrasound equipment [6]. To identify and solve ergonomic problems is a matter which concerns all levels in an organisation.

So far, sonographers’ perceptions of ergonomic problems at work has mostly been researched in quantitative studies [1, 6, 22]. There is a need of experience based research to deepen the knowledge about how sonographers perceive ergonomic problems at work. A qualitative study design is recommended when experience based knowledge is sought [23]. So far to our knowledge only a few qualitative studies have been performed in this area [16, 24].

The aim of this study was, therefore, to assess sonographers’ perceptions of these issues. The specific research questions addressed were: 1) What are the perceived ergonomic problems in echocardiography and vascular sonography? and 2) How can the working situation be improved ergonomically?

## Methods

### Study design and procedure

A qualitative study was performed to ascertain sonographers’ perceptions and characteristics of their work [23]. The first author contacted the heads of seven clinical physiology and cardiology departments in hospitals in the south of Sweden to present the study. The sonographers interested in participating and fulfilling the inclusion criteria were listed, and a time was booked for a 1- h qualitative interview. The interviews took place at the sonographer’s workplace in a separate room. The interviews were recorded and transcribed. Pre-interviews and two pilot interviews, included in the study, were performed, and a number of minor corrections were made to the interview guide.

### Study participants

The sonographers were recruited from clinical physiology and cardiology departments in the south of Sweden. The participants were selected according to the following inclusion criteria: woman, interest in participating, and variation in age, seniority in sonography, workplace, examinations performed and musculoskeletal disorders. We thus attempted to enrol as varied a study group as possible, with a wide range of perceptions. A total of 22 female sonographers expressed interest in participating, and these formed the study group. The mean age was 45 years (24–59 years) and mean seniority in sonography was 13.5 years (0.5–36 years). All except one performed echocardiography, and twelve performed both vascular and echocardiographic examinations. Thirteen worked full-time and all worked at least 20 h per week.

**Fig. 1:**
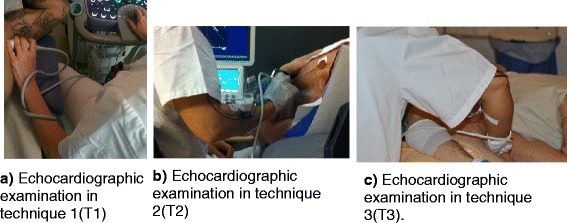
Pictures showing the three main echocardiographic examination techniques: Text to each picture: **a** T1, patient facing examiner, transducer in left hand, **b** T2, patient facing examiner, transducer in right hand, and **c** T3, patient facing away from examiner transducer in right hand

Three main working techniques are employed and taught in echocardiography, depending on local hospital practice. In technique 1 (denoted T1) the patient is facing the examiner, who holds the transducer in the left hand (Fig. 1a). In technique 2 (T2), the patient is also facing examiner, but the transducer is held in the right hand (Fig. 1b). In the third technique (T3), the patient faces away from the examiner, and the transducer is held in the right hand (Fig. 1c). T1 was most common (*N* = 9) followed by T3 (*N* = 8). In vascular scanning the technique varies depending on the type of examination.

### Data collection

A semi-structured interview guide was developed to address the research questions. The opening question was: “How do you perceive your sonographic work?” The interview guide contained open questions concerning ergonomic, psychosocial and organisational problems at work, as well as possible solutions to these problems and improvement strategies. Both positive and negative factors were elicited. The interview was performed as an open conversation, and the interviewer used follow-up questions to ensure that the research questions were answered in depth. The data were collected over a period of 9 months.

### Analysis

This paper focuses on ergonomic problems and improvement strategies. Content analysis was used to analyse the transcribed interviews [25, 26]. First, the interviews were read through several times by the first author to obtain a sense of the whole and to identify meaning units, i.e. relevant quotes related to the aim and research questions [27]. The first and second author then read and discussed the meaning units, condensed and coded them, and finally grouped them into categories according to each research question [25]. The categories and subcategories are described in the result together with quotations from the participants (indicated by a participant number).

## Results

The result showed that most of the sonographers perceived their work as stimulating, including contact with the patients, but found it physically exhausting. Four categories emerged from the first research question: I) working postures and type of examination, II) equipment including physical factors (light, noise and ventilation), III) the patient’s physique and health, and IV) the sonographer’s working experience and knowledge (Table 1). The analysis of the second research question revealed several ergonomic improvement strategies, which were grouped into three categories: I) working tasks and postures, II) equipment and physical factors, and III) professional working strategies (Table 2).

**Table 1:** The table shows: Ergonomic problems divided into categories and subcategories

Ergonomic problems				
Categories	Working posture and type of examination	Equipment and physical factors	The patient’s physique and health	Work experience and knowledge
Subcategories	-Echocardiography -Vascular examinations -Bedside examination	-Ultrasonic device -Examination room including table and equipment to assist patients -Physical factors including light, noise and ventilation	-Patient constitution -Inpatients	-Lack of skills

**Table 2:** The table shows: Ergonomic improvement strategies divided into categories and subcategories

Ergonomic improvement strategies			
Categories	Working tasks and postures	Equipment and physical factors	Professional working strategies
Subcategories	-Strategies for good working postures	-Ultrasound unit -Robot-assisted transducer -Physical factors: temperature, noise and lighting	-Practice and skill -Analysis -Patient handling

### Ergonomic problems

I. Working postures and type of examination
  The sonographers perceived that the type of examination influenced their working posture to a high degree, with less variation in posture in echocardiography compared to vascular examinations. Echocardiography was perceived to be extremely physically demanding as several measurements were required.
  “I’m tired because I’ve done a lot of echocardiographs this week, and then I feel more tired.” (9)
  Regardless of the technique used for echocardiography (T1–T3), the handling of the transducer was perceived as static and strenuous.
  “It’s really only in heart examinations that the hand is still for so long.” (20)
  If the patient lay on the table facing away (Fig. 1c, T3), the examiner had to stretch her arm to obtain good images, particularly if the patient was corpulent, or was lying far away on the table.
  “If the patient is too far away on the table, I have to stretch further.” (8)
  When mapping veins the examiner occasionally sat on the floor or on a chair with no possibility to rest the hand holding the transducer.
  “You map out the veins in the patient’s arms, sometimes both, so you don’t have any support for your own arm, that’s the problem.” (7)
  The hand managing the control panel was stretched out several times to reach the touch screen, especially in echocardiography.
  In bedside examinations the ultrasound equipment is taken to the ward, implying poor ergonomic postures and insufficient room for the examination.
  “We take the equipment to the ward and do the ultrasound exam on patients that can’t be moved. You have to position the equipment to accommodate the patient in bed. We sometimes stand up as there isn’t much room.” (16)
  Obtaining high-quality images was perceived as a higher priority than adjusting the equipment to achieve a comfortable working posture.
II. Equipment and physical factors
  Some sonographers reported ergonomic problems associated with the control panel, keyboard and screen, for example, the buttons were not within comfortable reach and the screen was not sufficiently physically adjustable. The leg space in front of the ultrasound unit was too narrow, the transducer uncomfortable to handle, and the cable was heavy. The transducer was sometimes difficult to grip, especially if it became slippery.
  “The transducers are hopeless… you use this gel and it makes things slippery. They’re quite heavy, and the cable makes them heavier at the end, they aren’t very easy to grip, they’re made of hard smooth plastic.” (7)
  A few of the echocardiographers had experience of a robot-assisted transducer attached to an arm, but perceived it difficult to handle.
  “Instead of holding the transducer with your hand, you’re supposed to manoeuver it with a little joystick. I’ve tried it, and it’s definitely not easy.” (9)
  The examination room was perceived to be too small, especially when examining an inpatient in bed. Sometimes, the ultrasound unit had to be moved to make room, which meant extra problems.
  “Patients who are brought in their beds—they’re so big these days—so for there to be enough room for the patient, my chair and my equipment, as well as the desk, I think the rooms are far too small.” (9)
  Lack of a patient lift also made it difficult to move the patient onto the examination table. Some of the sonographers also perceived that the tables were not sufficiently adjustable for different examinations and patients. The ultrasound unit gave off heat, even when additional cooling units had been fitted.
  “When the equipment has been on all day, it feels like a sauna in here.” (2)
  Exposure to noise was also perceived as a problem. The sonographers were used to the fans and were not aware of the noise until they turned the equipment off or left the room.
  “The fans make a noise all the time, but you get used to it… You only think about it when you turn the machine off. Then it’s quiet.” (9)
  Some of the sonographers reported eyestrain and headaches due to poor lighting in the examination room. To avoid glare on the screen the ceiling light was only switched on when the patient entered the room. Daylight sometimes came in through a slit in the curtain, which caused irritation. The same problem occurred when examining a patient on the ward, as there was no means of darkening the room.
  “You get mentally tired, and your eyes get tired… I don’t want all the ceiling lights on as then I have to squint… the lights mustn’t be visible on the screen… I mean, there mustn’t be reflections.” (8)
III. The patient’s physique and health
  The participants reported that the patient’s physique and health often had negative effects on their work load, their posture and the ability to obtain good images. Corpulent patients and patients with lung disease often meant longer examinations (transducer time) due to the poor quality of the images, and the need to press the transducer harder to obtain good images.
  “So fat that you have to almost lie over the patient to get around… to get close to the heart. There’s a lot of fat in the way.” (18)
  Slim patients were also a problem as their ribs were an obstacle to the examination of the heart. When inpatients were brought to the examination room the sonographers sometimes perceived a high workload and apprehensive.
  “Yes, because the patients are often very ill, and some of them aren’t very mobile either, and that means heavy work.” (7)
  Patients in intensive care were sometimes in a respirator, which hampered communication. The priority was high to make the patient comfortable and safe. If the patient was in pain the examination was completed as quickly as possible to reduce discomfort.
  If the inpatient arrived in a wheelchair and was too heavy to move, the examination was performed with the patient sitting in the wheelchair, which was perceived as demanding by the sonographer, who had to adjust her working posture, i.e. bend forward.
  “Sometimes we do it (the examination) in a wheelchair. Not so many heart exams, but there are lots of vascular patients who come in a wheelchair, and they’re so heavy you can’t lift them.” (10)
IV. Work experience and knowledge
  Lack of experience led to extended transducer time and too hard pressure in a static position.
  “That’s probably right… a common beginner’s problem is that you press really hard.” (9)
  “If you’re not an experienced sonographer, you tend to keep the transducer still for a long time until you get a good picture.” (2)
  Lack of knowledge and experience also implied stress, such as tense shoulders and forgetting the physical risks. On the other hand, a high degree of skill and experience often meant examining more patients per day.
  “You need a lot of knowledge to do an ultrasound exam. Then the pictures have to be good enough to interpret, to resolve the question on the referral.” (20)
  Obtaining images of high quality was given higher priority than good ergonomics.

### Ergonomic improvement strategies

I. Working tasks and postures
  The strategy employed to vary posture was to swap hands. Some of the sonographers changed the hand holding the transducer during some vascular examinations, but not in echocardiography.
  “I use my left hand as much (as my right) to hold the transducer in all examinations except the heart.” (13)
  Another measure employed was to adjust the position of the equipment and the patient before each examination.
  “I make sure the patient moves until I get a good working position.” (4)
  Standing up during scanning also made it possible to change posture and was also perceived as a relaxed position when managing the control panel and handling the transducer. Resting the transducer hand on the table, on the armrest or on the patient, and the other hand on the control panel were other strategies.
  “Sometimes you can stand up to do the exam, then it’s easier to get to, or around the patient, and relieve the strain in another way.” (2)
  A physiotherapist had instructed them on how to adjust the screen in order to reduce the amount they had to turn their head during scanning. Another way of avoiding strenuous postures was to work in a team of two sonographers, i.e. one managed the control panel and screen, while the other handled the transducer.
  “Two sonographers. I think that’s really good—we work in similar ways and it works really well.” (8)
  Vascular examinations were perceived as less physically tiring than echocardiography, as they were more varied and involved less static positions. To ensure variation in workload, the working day was divided into four sessions, so that echocardiography was performed in one session, after which other tasks were carried out.
  “We feel much better when we divide the day into four sessions.” (8)
  When seated and using techniques (T1 and T2)) for echocardiography, the sonographer tried to sit turned towards the patient to avoid having a bent wrist when handling the transducer.
  “I sit turned more towards the patient if he or she is lying down, so that I don’t have to bend my wrist, so I can keep it straight.” (16)
  Scanning facing the patient was perceived as preferable as it was possible to rest the arm on the armrest. When the patient was lying down facing away from the sonographer (T3), it was necessary for the examiner to stretch their arm more.
  “We have a chair with armrests that you can rest your arm on. What’s better with the first technique… is that I don’t have to stretch as much to get where I need to go.” (5)
  Standing and using the weight of their body during echocardiographic examination was a strategy used to facilitate the transducer projection, especially in corpulent or overweight patients, regardless of the working technique.
II. Equipment and physical factors
  An easily and highly adjustable control panel with touch buttons that could be positioned to minimize arm extension and finger pressure was considered a desirable improvement to ultrasound equipment. Likewise, a more adjustable screen would facilitate a comfortable neck position. Sufficient space for the legs when seated was another suggested improvement.
  “It must be possible to move the screen on the ultrasound equipment, a better arm, so I can raise and lower it and turn it how I want to. Buttons that are very close to me so I don’t need to reach out my arm, with touch buttons or buttons that are easy to press. A keyboard that can be moved up and down and sideways, that I can pull. Lots of space so you can get your legs in under the equipment. An ultrasound unit that’s easy to move.” (7)
  A light, wireless transducer with a comfortable grip and a cover to avoid gel smears were also suggested. A cable hook attached under the equipment was perceived to facilitate handling of the transducer.
  The use of a robot-assisted transducer in echocardiography was perceived to reduce awkward postures, manual pressure and pain due to strained positions. Continued training was recommended to improve handling.
  “Then I can steer it so that it moves towards the patient’s chest, and I can make most of the small movements that a hand can do with the remotely controlled control panel. I wouldn’t have to sit and press (the transducer) on the patient myself—it would do the heavy work. I can steer it now without thinking, it comes automatically, just like when I’m using my hand.” (18)
  An adjustable examination table, electrically controlled with a foot pedal, would facilitate positioning the patient and adjusting the height during the examination. A more adaptable table would facilitate performing different examinations using the same table. Also, a table resembling a dentist’s chair was suggested. Technical developments and improvements of the ultrasound unit have led to more distinct images suitable for computerized image analysis. A computer workstation was also perceived to be a better ergonomic alternative for reviewing the images, where the keyboard, screen and artificial light were adjustable and daylight could sometimes be used.
  “It’s better for me—I know some people stay at the ultrasound unit, but then you have to click on every image, and on the computer I can scroll.” (6)
  Larger examination rooms with automatic door openers would facilitate bringing a bed into the room, and examining the patient without having to move the table or the equipment. Two screens were perceived to facilitate the examination when two sonographers worked together. Ergonomic aids such as a sliding sheet, a turntable and patient lift were perceived useful if located close at hand. The noise level was perceived to have improved, as the newer ultrasound units were quieter and silencers had been fitted.
  “The machines have become quieter… so that’s better.” (7)
  “We have textiles, curtains… so it doesn’t echo so much.” (8)
  The air-conditioning system was perceived to be sufficiently adjustable in some workplaces. In others, additional cooling units had been installed to improve the ventilation, especially in smaller rooms.
  “Then there’s the temperature, yes, we have air conditioning in the room, so we can raise and lower the temperature, that’s good.” (18)
  The participants perceived that the possibility of adjusting the artificial light with a dimmer, and low-glare screens had improved the lighting conditions. These made it possible to increase the level of artificial lighting in the room.
  “The lights in the room have dimmers, so that can be adjusted.” (13)
  “The computer screens are better these days, there isn’t so much reflection in them, so I decided to turn the lights on, I can still see just as well.” (7)
  Spectacles for computer work were reported to facilitate scanning and computer work.
III. Professional working strategies
  Most of the sonographers perceived that achieving good images depended on practice to improve skills and increase experience. Cooperation with the doctor when reviewing the images provided an opportunity to improve knowledge and understanding. The broader the skills of the sonographer, the greater the possibility to alternate between different tasks and kinds of examinations.
  “The more (sonographers) who know how to do everything, the better it is. It’s also better for that person ergonomically.” (21)
  In some workplaces a resource person was available to assist if a colleague was delayed or a patient was difficult to examine.
  “I try to help out if I see that someone from sonography is late.” (12)
  Being well-accustomed with the equipment was perceived as improving the likelihood of a comfortable scanning posture, especially handling the transducer with less pressure.
  “No, when I’ve got the image I need, I try to relax my hand and find it again. I’ve been doing this so long it’s no problem.” (7)
  Some employed a strategy that involved shortening the scanning period, i.e. the transducer time, while others believed that taking more images would facilitate the analysis.
  “The work afterwards takes longer. I try to do as short examinations as possible to spare my body.” (1)
  Depending on local practices, some sonographers analysed the images directly on the unit after scanning, while others did it afterwards at a separate workstation. The latter method was perceived as shortening the time spent at the ultrasound unit, and providing the possibility to change posture.
  Consultation with a more experienced colleague was recommended if the recommended scanning time was exceeded. During prolonged examinations, e.g. mapping of veins, a team of two examiners was stated to be good practice, which also shortened the total examination time.
  It was also stated that preparing for the examination by checking the images from previous examinations was a good strategy. Seriously ill or unstable patients were examined in bed and, if necessary, a colleague or a member of staff from the ward was asked to assist. Scanning facing the patient facilitated observation of the patient’s face.
  “I always ask the patient if he or she can stand, and if they can move, and if they can’t, I fetch someone who can help me with the patient, so I don’t try to move them by myself.” (2)

## Discussion

### Methodological considerations

The selection of the sonographers included in this study was based on several inclusion criteria to ensure a variety of perceptions [25]. One of the inclusion criteria was interest in the study, which may have led to a selection bias, in that those with a high workload and/or musculoskeletal pain may have participated to a higher extent than others [28]. The interviews were planned by the head of department. One hour was allowed for each interview, and the interviews were performed during normal working hours at the sonographer’s place of work. The interview guide was followed, but was flexible regarding the order in which the questions were asked. Twenty-two sonographers participated and saturation was achieved concerning the inclusion criteria [29].

The interview questions were developed by the authors together with two researchers well-versed in the research questions and the aim of the study. The credibility of the results may have been increased by the fact that the first author had performed pre- and pilot interviews, and had a prior understanding of the sonographers’ working situation, which facilitated the development of the interview guide [23]. The second author, who was experienced in content analysis, also checked all the meaning units identified, and the authors discussed the development of the results continuously, which may also have increased the credibility. During analysis, the authors took into consideration the recommendations in the consolidated criteria for reporting qualitative research (COREQ) [29].

The results were based on the participants’ unique perceptions, which allowed the researchers to gain a deeper understanding of the sonographers’ working situation. This was a suitable method of obtaining information, i.e. giving the participants a voice [23]. The results should, therefore, be valid for all medical staff who use ultrasound as a diagnostic tool [30].

### Discussion of the results

The sonographers were aware of the ergonomic problems in their working environment, but these were usually not prioritized which is in line with previous results [6]. Thus, many suggestions were made for improvements to the working situation.

Echocardiography was considered physically demanding, regardless of the technique used, as the examination was performed with little variation in working posture and required simultaneous handling of the transducer and the control panel to obtain the required images. Carrying out the examination facing the patient (T1, T2) helped keep the transducer arm less abducted, which is in line with recommendations in previous research also put forward in the industry standards [21, 31]. Being able to use both hands on either the transducer or the control panel would be even better, which is feasible in T1 and T2. Ambidextrous scanning in echocardiography should be encouraged, and has been suggested in a previous study [8]. We recommend two examiners especially in more complicated examinations, to avoid delay and to shorten the total examination time, which may be a feasible and cost-effective strategy and also a good practice. Moreover, it may reduce the stress for the patients, especially those who are in pain. Such a routine requires the availability of qualified staffs which is sometimes a problem.

The sonographers’ perceptions of how the equipment could be optimized to make it more individually adjustable should be acknowledged and supported. This is in line with the accommodation to user anthropometrics described by Park et al. [32]. Equipment adjustable to suit the anthropometrics of the 5^th^ to the 95^th^ percentiles of the population is recommended in the industry standards [21]. Inappropriate transducer design has been noted previously [33]. In this study, a wireless transducer was suggested, but such a transducer is not accessible. Lightweight, neutral grip and flexible cables are recommended in the standards, but not a wireless transducer [4, 21].

An articulating support arm system for left-hand scanning was developed and tested in echocardiography to reduce the gripping of the transducer in strenuous and static postures, [16] which is in line with the development of a robotic arm [20]. Some of the participants in our study had positive experiences of the robot-assisted transducer, designed for tele sonography [19, 20], as no handgrip nor manual pressure was required. This device needs to be further introduced and tested to facilitate the implementation in echocardiography as an ergonomic solution, especially for corpulent patients where higher grip forces are needed to achieve the images [8]. A deeper cooperation between technical experts and medical expertise, i.e. the sonographers, might facilitate such an implementation. The development of a standardized report system in echocardiographic imaging is an example of how technical representatives participated together with the expertise in cardiovascular imaging [34, 35].

Some of the sonographers in our study suggested a limitation on transducer time, and image analysis at a separate computer workstation, where individual adjustment, avoidance of noise and better visual conditions are possible. These aspects are prerequisites for good work conditions when working with computers and standard in some of the workplaces [36, 37]. The examination room, including equipment and light, is designed primarily for sonography. To facilitate positioning of the patient on the table before each examination, efforts should be devoted to developing the equipment and the examination room so as to be more adaptable to the patient’s physique and health.

Several aspects of sonographers’ working situation must be improved in the future. These include ergonomic aids, scheduling and optimization of workplaces used for scanning. Scheduling of varied examinations and tasks, requires both access to different work task and broad skills. It is also important to motivate sonographers to take an active part in the ergonomic aspects of their work. Further research is required on ways in which this can be achieved.

## Conclusions

The sonographers perceived their work to be stimulating but physically exhausting. They were aware of the ergonomic problems associated with the patient’s poor health. However, the patient’s comfort and obtaining good images were often prioritized to the detriment of working posture. Ergonomic improvements were suggested, such as reducing the manual handling of the transducer, optimizing equipment adjustability and taking the patient’s physique and health into account. As some examinations were perceived more difficult than others, variation in examinations was suggested which, however, also requires broader skills.

## Abbreviations

T1: Technique one
T2: Technique two
T3: Technique three
